# Progress in Surgery

**Published:** 1900-07-07

**Authors:** 


### PROGRESS IN SURGERY.

### HERNIA.

**Bilocular Hernia.**—B. G. A. Moynihan[1] discussed exhaustively the anatomy of this form of hernia in the Arris and Gale Lectures. He thinks that the title bilocular hernia may with advantage be used to include the various forms of interstitial hernia, viz., the "pro-peritoneal herniæ," inguinal or crural, and the interstitial with a sac lying either (*a*) between the internal and external oblique muscles, or (*b*) between the external oblique and the skin. The apt terms "intra-parietal," "inter-parietal," and "extra-parietal" have been applied to the three forms of hernia by Rose and Carless. In each form the hernial sac has two loculi, one of which may be crural. Probably the bilocular form of hydrocele is very closely related developmentally to these forms of hernia.

In properitoneal hernia Moynihan describes three positions which the inner sac may occupy, viz: (1) it may pass to the outer and upper sides of the internal ring; (2) it may pass directly backwards and occupy the inner part of the iliac fossa near the pelvic brim; or (3) it may pass downwards and inwards into the true pelvis, lying by the side or in front of the bladder. The outer sac may be within the inguinal canal, or have reached the scrotum, or in rare cases is interstitial. The right side is more frequently affected than the left, and in a striking proportion of cases, viz., in 23 out of a total of 59, an imperfect descent of the testis has been recorded. It is interesting to note in this connection that normally the descent of the testicle, and the closure of the processus vaginalis, is later on the right side than on the left. Most frequently the sac is of the congenital variety. Ætiologically the hernia seems to arise most frequently as the result of some congenital abnormality, *e.g.*, peritoneal pouching. In some cases "reduction in mass" may be the responsible factor, and in a few cases the inner sac may be formed first, the external sac then being a secondary diverticulum. If the descent of a hernia is opposed at the external ring from contraction, or by an obstructing testicle, or an ill-fitting truss, the formation of a properitoneal sac will be favoured. In 67 per cent. of the cases of interstitial hernia the testicle is incompletely descended, and the descending sac rarely extends lower than just outside the external ring. This form of hernia is relatively more frequent in females. Wasting, or congenital defect of the abdominal parietes below the level of the anterior spine, is regarded by Macready as an important predisposing cause, but Moynihan thinks this is more likely a secondary effect of the pressure of the hernia.

Properitoneal hernias are, with the rarest exceptions, only recognised when strangulation occurs, and in many cases there has been apparent reduction, but the symptoms have persisted. Moynihan's impressions, gathered in an examination of specimens in the London museums, is that the very great majority of examples of reduction in mass are really cases of properitoneal hernia in which the contents have been reduced from one sac into the other. Van Buren Knott[2] writes a paper on a case of properitoneal and interstitial hernia. His classification is the same as that given above, but he omits the variety in which the sac lies between the skin and the external oblique. The properitoneal form is considered much less frequent, and more difficult to recognise than the interstitial. Knott thinks all these hernias should be operated upon whether strangulated or not. L. Grant Baldwin[3] reports a case of properitoneal hernia. The patient, a woman of 38, was admitted to hospital for obstinate vomiting of three days' duration, epigastric pain, and obstruction of the bowels for five days, and was regarded as a case of "intestinal obstruction." Six hours later a small lump was felt in the inguinal region, about an inch and a-half above Poupart's ligament. An operation the next day showed a hernia the size of half a walnut, a partial enterocele, the sac being properitoneal and passing inwards almost to the border of the rectus muscle. No mention is made of a second pouch in the inguinal canal. If one was not present the hernia cannot be called "bilocular."

[1] Lancet, Feb., 1900. [2] Vide The Post-Graduate, Jan., 1900. [3] Med. Rec., Jan., 1900.

### OBSTETRICS.

**Puerperal Eclampsia.**—With reference to the etiology of this dangerous affection Easterbrook[1] quotes an interesting case of a woman who, with a family history of insanity, showed at different times chorea (at 14 and at 22, during pregnancy), status epilepticus, and puerperal insanity. At 15 she had melancholia, with acute mania two months later; at 16 and 19, mania; at 22, puerperal mania, followed by chorea six weeks later; mania at two subsequent pregnancies.

Easterbrook holds that each of these states has a common site in the cerebral cortex (? pre-Rolandic area); that they are functional neuroses, and depend upon molecular rather than gross structural changes. The toxins found he holds to be the outcome, and not the cause, of the morbid nervous metabolism. This is supported by their incidence during the developmental and maturative periods of life.

**Prevention.**—Charles, of Liége,[2] considers that the mortality of this disease may be lessened by carefully watching the urine of pregnant women, and giving appropriate medical treatment of albuminuria when present by means of purgatives, milk diet, with Vichy water, &c., bromides and chloral, especially during the seventh and eighth months. When convulsions have occurred, he urges dilatation of the cervix and premature delivery, holding that this is not a dangerous procedure, as many have insisted, for that the cervix is not nearly so sensitive as has been urged. Chloroform should be used freely.

Coudray,[3] however, considers that induction of labour must not be attempted unless the patient has been placed under an appropriate diet previously; this diet to consist exclusively of milk and kefir. When after a week or so the albumen has been found to remain stationary, or to be growing worse, then induction is indicated if the uterus is well developed. If this is not so, then the diet must be continued. If the fœtus dies, then the albuminuria of pregnancy ceases.

Davis[4] holds that convulsions can be controlled by chloroform, large doses of morphine, veratrum viride, or nitrite of amyl. The albumin in urine, he says, is only of importance when large in amount and associated with kidney *débris*. Prevention may be sought by diet of milk, bread and butter, green vegetables, and fruit, with much pure soft water. Fresh air and gentle exercise is of importance, but no alcohol should be allowed.

Norris[5] regards obstinate neuralgia, irritable temper, excessive vomiting, marked salivation, coated tongue, hebetude, and signs of inactivity of the liver generally as an indication for treatment in cases of albuminuria. Toxin formation is to be prevented by diet; the excretory organs must be aided to excrete them. The colon should be irrigated daily with two gallons of salt solution. Chloroform is better than morphine for controlling the convulsions.

Reynolds[6] urges diuresis, catharsis, and diaphoresis, rest in bed, milk diet, and mild sedatives (bromides). Immediate delivery under ether after one convulsion. He prefers chloral to morphine for controlling convulsions.

Mackie[7] gives a case where a woman had a period of stupor seven days after her second and third parturition, without previous signs of any trouble. At the fourth parturition she showed some œdema of the ankles, the urine contained much albumen. Eight hours after the delivery convulsions set in, rapidly recurring. ℥ix. of blood were drawn, saline injections into the rectum, hot fomentations to abdomen, gradual recovery with careful dietary. Seven days later apathy set in, and death ensued with symptoms of cardiac failure.

Coirigan[8] gives an instance of eclampsia in a girl of 15, the convulsions occurring before delivery. Great loss of blood, recovery after warmth, chloroform, ergot and brandy.

Spurr[9] quotes three cases, and notes that chloroform is to be relied upon to check convulsions, but it often has to be pushed to the full surgical degree. He has full confidence in chloral hydrate and pot. bromide. In one case the albumen was detected early and treated, and although eclampsia occurred yet it was not so severe. All three patients did well.

**Fibroids Complicating Pregnancy.**—Doléris[10] recommends cervical pediculated fibroids to be removed. Very prominent or pediculated subperitoneal fibroids on fundus or body must be removed "on the slightest sign of the pregnancy being interrupted or danger appearing." Rapid increase is an indication for removal. With multiple and large intra-mural fibroids pregnancy should be considered of little import. Early operation shows least mortality, while the longer such a pregnancy exists the more danger to the patient. A simple intra-mural fibroid of moderate size and giving no trouble should be left alone.

Van Tussenbroek[11] gives a case of the often denied ovarian pregnancy. He examined a case of Kouwer's in 1893, and arrives at the conclusion that the case was a genuine one of a pregnant graafian follicle. No adhesions existed between ovary and tube, and the mesovarium was considerable. In the follicle a fœtus 12 mm. long was found, surrounded by amnion and chorion. No decidual tissue was found, but syncytium was present, proving that this structure is fœtal only, and has no connection with the uterine epithelium. The fact that no decidua was found militates against the theory held by Webster that an ovum can only be grafted where the tissues can undergo decidual changes.

Foulis[12] shows that the umbilical cord has no covering of amnion at any period of its existence, but that the cord proper is always within the amniotic sac, and consists in a tubular prolongation of the somatopleure round the collapsed and shrivelled yolk sac. He showed micro-photographs of sections of the embryos of the deer and sheep. Before the cord had an existence as such, the amnion could be traced as an outgrowth from the side of the "bauchstiel" of His, and from the somatopleure round the large patent umbilical foramen. The "bauchstiel" is the forerunner of the umbilical cord; but to trace the formation of the latter from the former is difficult, without remembering the relation of the somatopleure to the amnion. Transverse sections of the "bauchsteil" showed that the amnion was the extension of the somatopleure folds, of which the bauchstiel mainly consisted. As the amniotic cavity becomes filled with fluid, the amnion is raised from the embryo, except around the umbilical cavity and the margins of the "bauchstiel." The shrinking of the yolk sac is coincident with the closing of the umbilicus. The somatic mesoblastic surfaces of somatopleure when in contact around the yolk sac adhere together, but at the distal end of the cord, and not at its proximal end. Thus the amnion does not cover the cord, but will cover the placenta on the fœtal surface, and can hence be stripped from it, but not from the cord.

**Interesting Cases.**—McCoy[12] quotes an interesting case of a woman, who, having a contracted pelvis, lost one child by craniotomy. She then underwent two successive Cesarean sections. After the first a sinus was left connected with uterine cavity. Twenty-four hours before menstrual blood appeared per vaginam, hæmorrhage appeared along the track. During the ensuing pregnancy this ceased.

Bar[13] quotes a case in which the uterus was ruptured during a shoulder presentation, but the peritoneum was stripped from the uterus without being ruptured. This was shown on abdominal section being undertaken. The space below the peritoneum was drained and packed with gauze through the uterine wound, and the patient did well. Budin, commenting on the case, quoted several others similar, and expected that many cases of recovery after rupture were due to this phenomenon which had not been verified.

An interesting case is communicated by Paterson.[14] A woman, who was suffering from acute pneumonia, with marked aortic and mitral stenosis, was delivered of a child at term, without having shown much previous discomfort from this considerable pathological condition. The patient died after delivery in extreme dyspnœa. Both kidneys were found diseased—one cystic and tuberculous, and the other cirrhotic.

Prokess[15] reports a case of Cesarean section on a moribund woman, who died eight hours after the successful removal of a living child. The uterus was found contracting vigorously during the operation. The child did well.

Keiffer[16] reports the case of a woman who in five pregnancies only had one full-term child. In the sixth gestation premature rupture of the membranes occurred, with loss of nearly all the liquor amnii. The pregnancy continued till the eighth month, when a well-formed child $4\frac{1}{2}$ lbs. was delivered. No "chorionic pouch" was found on examining the membranes.

Pochin[17] records a case of complete, and Wilson[17] a case of partial inversion of the uterus, both producing great collapse. Digital reposition, easy recovery.

Hugo Hübl[18] gives rules for the diagnosis of air emboli in placenta prævia. (1) By exclusion of other possible diagnosis, such as ruptures of cervix and uterus, death from anæmia, chloroform, thrombus, emboli, &c. (2) If there is present over the heart a "clucking" murmur, and if in that region there is a tympanitic or dulled tympanitic sound, then the clinical diagnosis of this affection is certain. The air enters because the pressure in the abdominal uterine vessels is suddenly diminished, and air is sucked into the patent vessels; also pressure in the uterus is increased, and air is pressed into the gaping vessels.

Tridoudain[19] has found that pregnancy depresses the superficial reflexes, but increases the deep ditto. The pupil shows a state similar to the Argyll-Robertson phenomenon. After labour the normal state returns in about ten days.

[1] Brit. Med. Jour., May 5. [2] Brit. Med. Jour., May 5. [3] Brit. Med. Jour., April 7. [4] Med. Rec., Jan. 13. [5] Idem. [6] Idem. [7] Brit. Med. Jour., Mar. 10. [8] Brit. Med. Jour., April 7. [9] Lancet, June 16. [10] Brit. Med. Jour., June 23. [11] Brit. Med. Jour., April 14. [12] Lancet, June 23. [13] Brit. Med. Jour., Feb. 17. [14] Brit. Med. Jour., Mar. 10. [15] Scottish Med. and Surg. Jour., Mar. [16] Brit. Med. Jour., Mar. 31. [17] Brit. Med. Jour., April 14. [18] Lancet, Feb. 10. [19] Brit. Med. Jour., April 7. [20] Med. Rec., Feb. 24. [21] Brit. Med. Jour., June 23. [22] Brit. Med. Jour., April 28.

